# Development and validation of a nomogram for predicting hemoptysis risk in patients with bronchiectasis: A study based on Random Forest algorithm

**DOI:** 10.1097/MD.0000000000050019

**Published:** 2026-07-31

**Authors:** Gengyan Zhang, Yan Chen, Ming Zhai, Lianbo Zhao

**Affiliations:** a Department of Respiratory and Critical Care Medicine, Mengcheng First People’s Hospital, Mengcheng, Anhui, China.

**Keywords:** bronchiectasis, decision curve analysis, hemoptysis, nomogram, prediction model, Random Forest

## Abstract

Hemoptysis is a severe and potentially life-threatening complication of bronchiectasis. There is currently a lack of reliable tools for the individualized prediction of hemoptysis risk in these patients. This study aimed to utilize a machine learning algorithm to develop and validate a nomogram for predicting the risk of hemoptysis in patients with bronchiectasis. This retrospective study enrolled 131 patients with bronchiectasis, who were randomly divided into a training set and an internal validation set in a 7:3 ratio. The Random Forest algorithm was employed, using mean decrease accuracy as the metric, to screen for important predictors from clinical variables. Subsequently, the selected variables were incorporated into a multivariate logistic regression model to construct the predictive nomogram. The model’s discrimination, calibration, and clinical utility were comprehensively evaluated using the receiver operating characteristic curve, calibration curve, decision curve analysis, and clinical impact curve. The top 10 important predictors identified by the Random Forest algorithm included body mass index, C-reactive protein, creatinine, age, urea, low-density lipoprotein, platelet count, total cholesterol, diabetes, and fasting plasma glucose. Multivariate logistic analysis confirmed that body mass index, C-reactive protein, creatinine, age, and urea were independent predictors of hemoptysis. After correcting the mismatched logistic regression parameters, the constructed nomogram still demonstrated excellent predictive performance in both the training and validation sets, with an area under the curve of 0.944 (95% confidence interval: 0.873–1.000) in the validation set. The calibration curve showed a high degree of consistency between predicted probabilities and observed outcomes. Decision curve analysis and the clinical impact curve further confirmed that the model provided significant net clinical benefit across a wide range of threshold probabilities. This study successfully developed and validated a nomogram that incorporates Random Forest-based variable screening. The model integrates 5 readily available clinical parameters to accurately and individually predict the risk of hemoptysis in patients with bronchiectasis, demonstrating good calibration and clinical utility. It serves as a valuable tool to assist clinicians in the early identification and management of high-risk patients.

## 1. Introduction

Bronchiectasis is a chronic respiratory disease characterized by abnormal and permanent dilation of the bronchi due to various etiologies. Its global disease burden is significantly underestimated, with both prevalence and mortality showing a marked upward trend.^[1]^ The clinical presentation of bronchiectasis is highly heterogeneous, commonly featuring symptoms, such as chronic cough, excessive sputum production, and recurrent respiratory infections, which severely impact patients’ quality of life and long-term prognosis.^[2]^ Among its complications, hemoptysis is one of the most severe and potentially life-threatening, occurring in up to 20% to 30% of patients during the disease course.^[3]^ Although most episodes of hemoptysis are self-limiting and involve small volumes of blood, approximately 4% to 8% of patients experience life-threatening massive hemoptysis, which carries a high mortality rate and poses a major clinical challenge.^[4]^

Current comprehensive management strategies for bronchiectasis, such as airway clearance techniques and long-term antibiotic therapy, primarily focus on reducing acute exacerbations and improving quality of life.^[5,6]^ However, there remains a lack of effective primary prevention strategies specifically targeting hemoptysis. Clinical management is largely reactive, involving interventions such as hemostatic agents, bronchial artery embolization, or emergency surgery after an episode has occurred.^[7,8]^ This passive approach stems from the absence of reliable tools for accurately and individually identifying patients at high risk of hemoptysis. The preemptive identification of high-risk individuals enables clinicians to implement more intensive monitoring, patient education, and preventive interventions, such as advance discussion of bronchial artery embolization protocols, which can significantly optimize individual patient management and improve clinical outcomes.^[9]^

In recent years, the application of machine learning in the medical field has provided powerful tools for addressing complex clinical prediction problems.^[10]^ Among various algorithms, Random Forest is highly regarded for its superior performance. It is capable of handling high-dimensional data, capturing complex nonlinear relationships and interactions among variables, and is inherently resistant to overfitting.^[11]^ More importantly, its provision of variable importance measures, such as mean decrease accuracy (MDA), offers an objective, data-driven method for identifying the most clinically relevant predictors from a large set of potential variables.^[12,13]^ This represents a considerable advantage over traditional variable selection strategies based on univariate analysis or researchers’ prior knowledge, significantly reducing selection bias.

Although some studies have attempted to identify risk factors associated with hemoptysis in bronchiectasis,^[14]^ existing prediction models exhibit notable limitations. Most studies rely solely on conventional logistic regression or Cox proportional hazards models, whose variable selection processes may overlook critical nonlinear predictors.^[15]^ Furthermore, many models have not undergone rigorous internal or external validation, casting doubt on their generalizability.^[16]^ Perhaps most importantly, few studies have thoroughly evaluated the clinical utility of these models. A robust predictive model should not only demonstrate good discrimination and calibration but also prove its value in clinical decision-making through tools such as decision curve analysis (DCA), which quantifies net benefit across various risk thresholds.^[17]^ The nomogram serves as a visualization tool that translates complex regression models into individual risk probabilities, greatly facilitating clinical application.^[18,19]^

Against this backdrop and to fill the aforementioned research gaps while addressing unmet clinical needs, the present study adopted the Random Forest algorithm, an advanced data mining technique, to objectively screen key predictive factors from multidimensional clinical data and construct a nomogram model for individualized prediction of hemoptysis risk in patients with bronchiectasis. The study comprehensively validated and evaluated the established model by assessing its discrimination and calibration performance and emphatically verified its clinical utility via DCA and clinical impact curve (CIC) analyses. This validated prediction tool provides robust evidence-based decision support for the refined clinical management and targeted prevention of hemoptysis in patients with bronchiectasis.

## 2. Methods

### 2.1. Study design and patient cohort

This was a retrospective cohort study that consecutively enrolled patients diagnosed with bronchiectasis in the Department of Respiratory and Critical Care Medicine of our hospital between January 2020 and December 2024. The diagnosis of bronchiectasis was confirmed by high-resolution computed tomography (HRCT) and independently verified by 2 experienced radiologists. Cohen’s Kappa statistical analysis was performed to evaluate the interobserver agreement of HRCT diagnostic results between the 2 radiologists, and a high level of diagnostic consistency was confirmed (Kappa = 0.89), indicating excellent inter-rater reliability for bronchiectasis diagnosis in this cohort. Inclusion criteria were age ≥ 18 years, meeting HRCT diagnostic criteria for bronchiectasis, and availability of complete clinical data. Exclusion criteria included coexisting active tuberculosis or lung cancer and hemoptysis attributable to other causes, such as pulmonary embolism, heart failure, or coagulation disorders. The study protocol was approved by the Institutional Ethics Committee of Mengcheng First People’s Hospital (Approval Code: MCHEC-20250812, Approval Date: August 12, 2025), and the requirement for informed consent was waived due to the retrospective nature of the study. The primary outcome was defined as a “major hemoptysis event,” characterized by either expectoration of ≥100 mL of blood in a single episode or a total volume of ≥500 mL of blood expectorated within a 24-hour period, whether occurring prior to admission or during hospitalization.

### 2.2. Data collection and variable definition

Baseline patient data were collected through the electronic medical record system. All baseline laboratory measurements were uniformly performed within the first 24 hours of patient admission to ensure the timeliness, uniformity, and comparability of the baseline data. The collected information included demographic and clinical characteristics, including age, sex, body mass index (BMI), history of hypertension, and diabetes mellitus, as well as laboratory parameters including complete blood count (encompassing white blood cell count, neutrophil count, lymphocyte count, platelet count, and hemoglobin), C-reactive protein (CRP), fasting plasma glucose, alanine aminotransferase, aspartate aminotransferase, total bilirubin, alkaline phosphatase, albumin, globulin, gamma-glutamyl transferase, creatinine, urea, triglycerides, total cholesterol, high-density lipoprotein, and low-density lipoprotein.

### 2.3. Variable screening and model building

All patients were randomly divided into a training set and an internal validation set at a ratio of 7:3. For variable screening, the Random Forest algorithm was employed in the training set to evaluate variable importance and identify the most relevant predictors for hemoptysis events. Using the randomForest package in R (available from the Comprehensive R Archive Network [CRAN]), a forest comprising 1000 decision trees was constructed. MDA was selected as the metric for assessing variable importance. This method, based on permutation tests, directly reflects each variable’s contribution to the model’s predictive accuracy and is considered more robust than Gini importance, as it is less influenced by variable type and scale. The MDA values were standardized and ranked. To balance model predictive performance, structural simplicity, and generalizability, multiple variable inclusion thresholds (top 5, top 10, and top 15 variables) were explored in the preliminary analysis. The results showed that the model incorporating the top 5 variables exhibited insufficient predictive efficacy due to limited predictive factors, while the top 15-variable model introduced redundant low-contribution variables, increased model complexity, and failed to achieve significant performance improvement. Ultimately, the top 10 most important variables with the highest standardized MDA values were retained, as this threshold yielded the optimal trade-off between predictive accuracy and model parsimony for subsequent modeling.

For model construction, the selected variables were incorporated into a multivariable logistic regression model to examine their independent associations with hemoptysis risk. All statistical parameters (β coefficient, standard error, *z* statistic, odds ratio (OR), 95% confidence interval [CI], and *P*-value) were strictly derived from the unified final regression model output in R software to avoid data mismatch and transcription errors. ORs and their 95% CIs were calculated. Based on the logistic regression results, a nomogram for predicting the risk of hemoptysis was constructed in R.

### 2.4. Model validation and evaluation

The nomogram model was evaluated and validated in both the training and validation sets using the following metrics: Discrimination: The receiver operating characteristic curve was plotted, and the area under the curve (AUC) was calculated to assess the model’s ability to distinguish between patients with and without hemoptysis. Calibration: A calibration curve was drawn to compare the predicted probability of hemoptysis against the actual observed frequency. The Hosmer–Lemeshow goodness-of-fit test was applied, and a *P*-value > .05, along with a calibration curve closely aligned with the 45-degree diagonal, indicated good agreement between the predicted and observed outcomes. Clinical utility: DCA curves were generated to quantify the net benefit of using the nomogram for clinical decision-making across various threshold probabilities. The net benefit of the model was compared against the “treat-all” and “treat-none” strategies. CIC curves were plotted to visually represent the number of individuals classified as high risk by the model at different risk thresholds, along with the corresponding number of true-positive cases. This illustrates the potential clinical impact of the model at the population level.

### 2.5. Statistical analysis

Continuous variables were expressed as mean ± standard deviation (if normally distributed) or median with interquartile range (if non-normally distributed). Group comparisons were performed using the Student’s *t* test or the Mann–Whitney *U* test, as appropriate. Categorical variables were summarized as frequency (percentage) and compared using the chi-square test or Fisher’s exact test. All statistical analyses were conducted using R software (version 4.2.2; R Foundation for Statistical Computing, Vienna, Austria). A two-sided *P*-value < .05 was considered statistically significant.

## 3. Results

### 3.1. Patient baseline characteristics

A total of 131 patients with bronchiectasis were ultimately included in this study, among whom 65 presented with hemoptysis. The patients were randomly allocated into a training set (n = 95) and a validation set (n = 36). Baseline demographic, clinical, and laboratory characteristics were well balanced between the 2 cohorts. No significant differences were observed in baseline characteristics between the 2 sets (all *P* > .05), indicating successful randomization (Table 1).

**Table 1:** Comparison of baseline characteristics between the training set and validation set.

Variables	Total (N = 131)	Testdata (N = 36)	Triandata (N = 95)	*P*
Age, median (Q1, Q3)	61.00 (51.50, 68.50)	60.50 (48.00, 68.25)	61.00 (53.00, 68.50)	.581
BMI, median (Q1, Q3)	37.70 (36.90, 41.20)	37.60 (36.90, 40.50)	37.70 (36.90, 41.20)	.951
WBC, median (Q1, Q3)	7.80 (6.20, 8.90)	7.60 (6.10, 8.80)	7.80 (6.35, 8.90)	.798
Lymphocyte, median (Q1, Q3)	2.00 (1.70, 2.50)	2.00 (1.60, 2.60)	2.00 (1.70, 2.50)	.926
Neutrophils, median (Q1, Q3)	4.70 (3.50, 5.95)	4.60 (3.60, 5.82)	4.80 (3.50, 6.00)	.861
Hemoglobin, median (Q1, Q3)	13.80 (12.90, 14.70)	14.00 (13.00, 15.05)	13.80 (12.80, 14.60)	.270
Platelet, median (Q1, Q3)	232.00 (186.00, 267.00)	249.50 (187.75, 268.50)	228.00 (186.00, 265.00)	.315
FPG, median (Q1, Q3)	6.44 (5.72, 8.07)	6.53 (5.52, 8.52)	6.44 (5.80, 7.78)	.885
ALT, median (Q1, Q3)	19.00 (13.00, 28.00)	18.00 (13.00, 28.75)	20.00 (13.50, 27.50)	.655
AST, median (Q1, Q3)	19.00 (15.00, 23.00)	18.50 (15.00, 24.25)	19.00 (15.00, 23.00)	.946
Total bilirubin, median (Q1, Q3)	0.40 (0.30, 0.60)	0.40 (0.30, 0.50)	0.40 (0.30, 0.60)	.154
Alkaline phosphatase, median (Q1, Q3)	82.00 (66.00, 99.00)	81.00 (63.75, 89.25)	85.00 (66.50, 103.00)	.120
Albumin, median (Q1, Q3)	3.80 (3.60, 4.00)	3.80 (3.70, 4.00)	3.80 (3.60, 4.00)	.390
Globulin, median (Q1, Q3)	32.00 (29.00, 35.00)	31.50 (29.00, 35.00)	33.00 (29.00, 35.00)	.490
GGT, median (Q1, Q3)	23.00 (18.00, 36.00)	23.50 (19.50, 40.50)	23.00 (17.00, 35.00)	.794
Creatinine, median (Q1, Q3)	74.26 (61.44, 90.61)	75.14 (60.78, 93.04)	74.26 (62.32, 90.17)	.707
BUN, median (Q1, Q3)	5.71 (4.28, 6.43)	5.71 (4.55, 6.34)	5.36 (4.28, 6.43)	.942
TG, median (Q1, Q3)	1.31 (0.89, 1.68)	1.29 (0.80, 1.59)	1.32 (0.90, 1.69)	.562
TC, median (Q1, Q3)	4.50 (3.83, 5.21)	4.46 (3.70, 5.40)	4.50 (3.87, 5.06)	.799
HDL, median (Q1, Q3)	1.16 (0.97, 1.40)	1.11 (0.96, 1.30)	1.19 (0.98, 1.42)	.295
LDL, median (Q1, Q3)	2.56 (2.09, 3.35)	2.81 (2.22, 3.37)	2.51 (2.07, 3.35)	.674
CRP, median (Q1, Q3)	23.27 (13.96, 28.83)	23.27 (14.74, 28.77)	23.27 (13.96, 28.83)	.717
Hemoptysis, n (%)				.736
No	66 (50.4)	19 (52.8)	47 (49.5)	
Yes	65 (49.6)	17 (47.2)	48 (50.5)	
Sex, n (%)				.445
Female	58 (44.3)	14 (38.9)	44 (46.3)	
Male	73 (55.7)	22 (61.1)	51 (53.7)	
Hypertension, n (%)				.718
No	94 (71.8)	25 (69.4)	69 (72.6)	
Yes	37 (28.2)	11 (30.6)	26 (27.4)	
Diabetes, n (%)				.460
No	97 (74.0)	25 (69.4)	72 (75.8)	
Yes	34 (26.0)	11 (30.6)	23 (24.2)	

ALT = alanine aminotransferase, AST = aspartate aminotransferase, BMI = body mass index, BUN = blood urea nitrogen, CRP = C-reactive protein, FPG = fasting plasma glucose, GGT = gamma-glutamyl transferase, HDL = high-density lipoprotein, LDL = low-density lipoprotein, TC = total cholesterol, TG = triglycerides.

### 3.2. Random Forest variable screening

**Figure 1.**
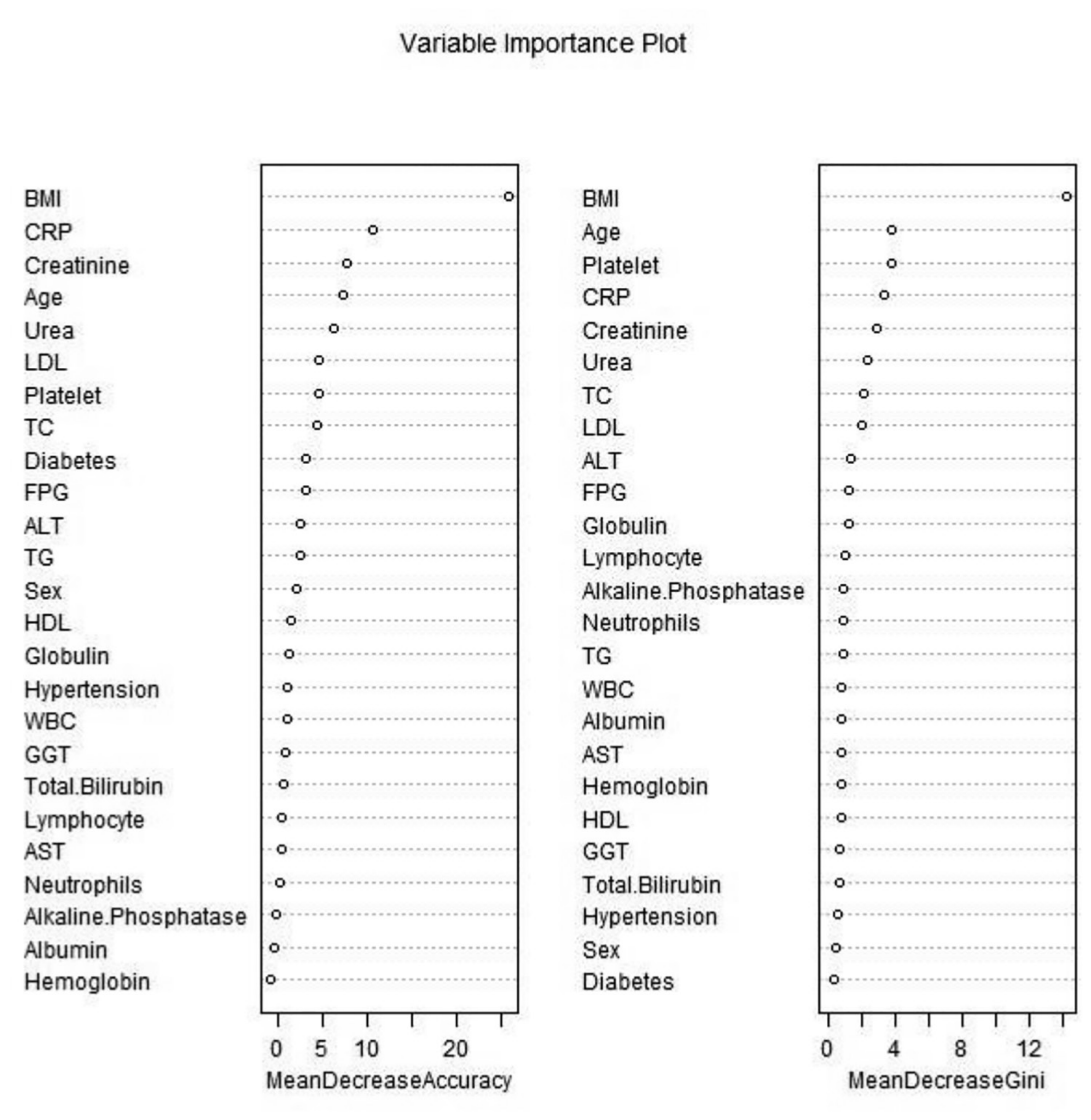
Predictor variable importance ranking based on Random Forest MDA. MDA quantifies the reduction in model predictive accuracy after permuting each variable; higher MDA values indicate greater predictive importance. The top 10 variables with the highest predictive contribution were retained for subsequent modeling. ALT = alanine aminotransferase, AST = aspartate aminotransferase, BMI = body mass index, CRP = C-reactive protein, FPG = fasting plasma glucose, GGT = gamma-glutamyl transferase, HDL = high-density lipoprotein, LDL = low-density lipoprotein, MDA = mean decrease accuracy, TC = total cholesterol, TG = triglycerides, WBC = white blood cell count.

To screen candidate predictive factors for major hemoptysis events, the Random Forest algorithm with the MDA metric was adopted for variable importance evaluation. Different from traditional univariate screening methods, this machine learning-based approach effectively captures nonlinear relationships between variables and avoids subjective screening bias, ensuring the objectivity and robustness of candidate variable selection. The algorithm identified the top 10 most important predictive variables and their relative importance rankings, as visualized in Figure 1. In descending order of predictive importance, the screened variables were BMI, CRP, creatinine, age, urea, low-density lipoprotein, platelet count, total cholesterol, diabetes, and fasting plasma glucose. These multidimensional indicators, covering physical baseline, inflammatory level, renal function, glycolipid metabolism, and underlying comorbidities, provided a comprehensive variable pool for subsequent independent predictor screening and model construction.

### 3.3. Multivariate logistic regression and nomogram construction

**Figure 2.**
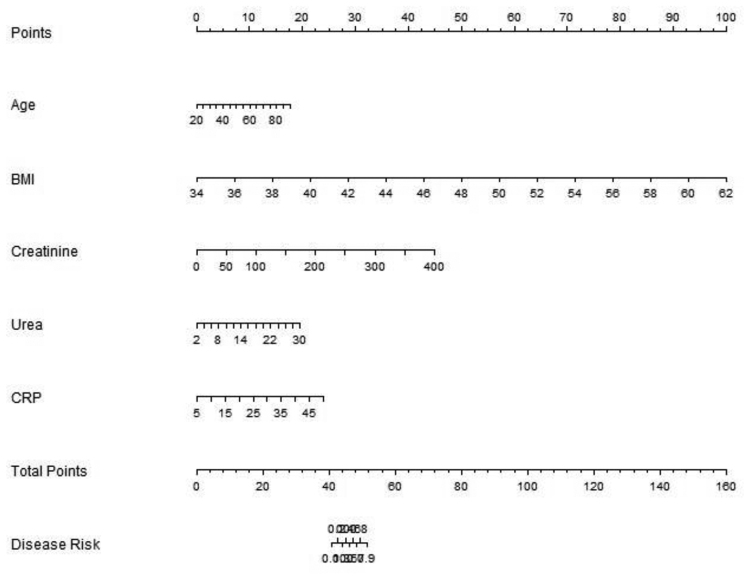
Nomogram predicting the individualized risk of hemoptysis in patients with bronchiectasis. Four independent clinical predictors (age, BMI, CRP, and creatinine) were integrated to calculate the total score and corresponding hemoptysis probability for each patient. BMI = body mass index, CRP = C-reactive protein.

The 10 selected variables were incorporated into a multivariate logistic regression model. After correcting the transcription errors in the logistic regression parameters, the results confirmed that 5 variables, including BMI, CRP, creatinine, age, and urea, were independent protective and risk factors for hemoptysis events (all *P* < .05), and all β values strictly matched the ORs calculated using the formula OR = exp(β) (Table 2). This parsimonious set of independent predictors is clinically reasonable and biologically plausible, covering multiple systemic dimensions closely related to respiratory disease progression and bleeding risk. Based on these 5 final predictors, a nomogram was developed to predict the risk of hemoptysis in patients with bronchiectasis (Fig. 2).

**Table 2:** Multivariate logistic regression analysis for predicting hemoptysis events in the training set.

Variables	β	Standard error	*z*	OR (95% CI)	*P*
Age	0.164	0.049	3.347	1.178 (1.078–1.287)	.030
BMI	1.288	0.449	2.868	3.624 (1.503–8.737)	.004
Platelet	−0.009	0.009	−1.010	0.991 (0.974–1.008)	.312
FPG	0.024	0.171	0.139	1.024 (0.732–1.432)	.889
Creatinine	0.144	0.030	4.800	1.155 (1.095–1.219)	.034
Urea	0.223	0.402	0.555	1.250 (1.169–1.347)	.023
TC	1.092	1.713	0.637	2.980 (0.104–85.531)	.524
LDL	−2.227	1.860	−1.197	0.108 (0.003–4.131)	.231
CRP	0.314	0.125	2.511	1.369 (1.071–1.750)	.012
Diabetes					
No				Reference	
Yes	0.796	0.498	1.599	2.216 (0.836–5.877)	.110

BMI = body mass index, CI = confidence interval, CRP = C-reactive protein, LDL = low-density lipoprotein, OR = odds ratio, TC = total cholesterol.

### 3.4. Model validation and evaluation

**Figure 3.**
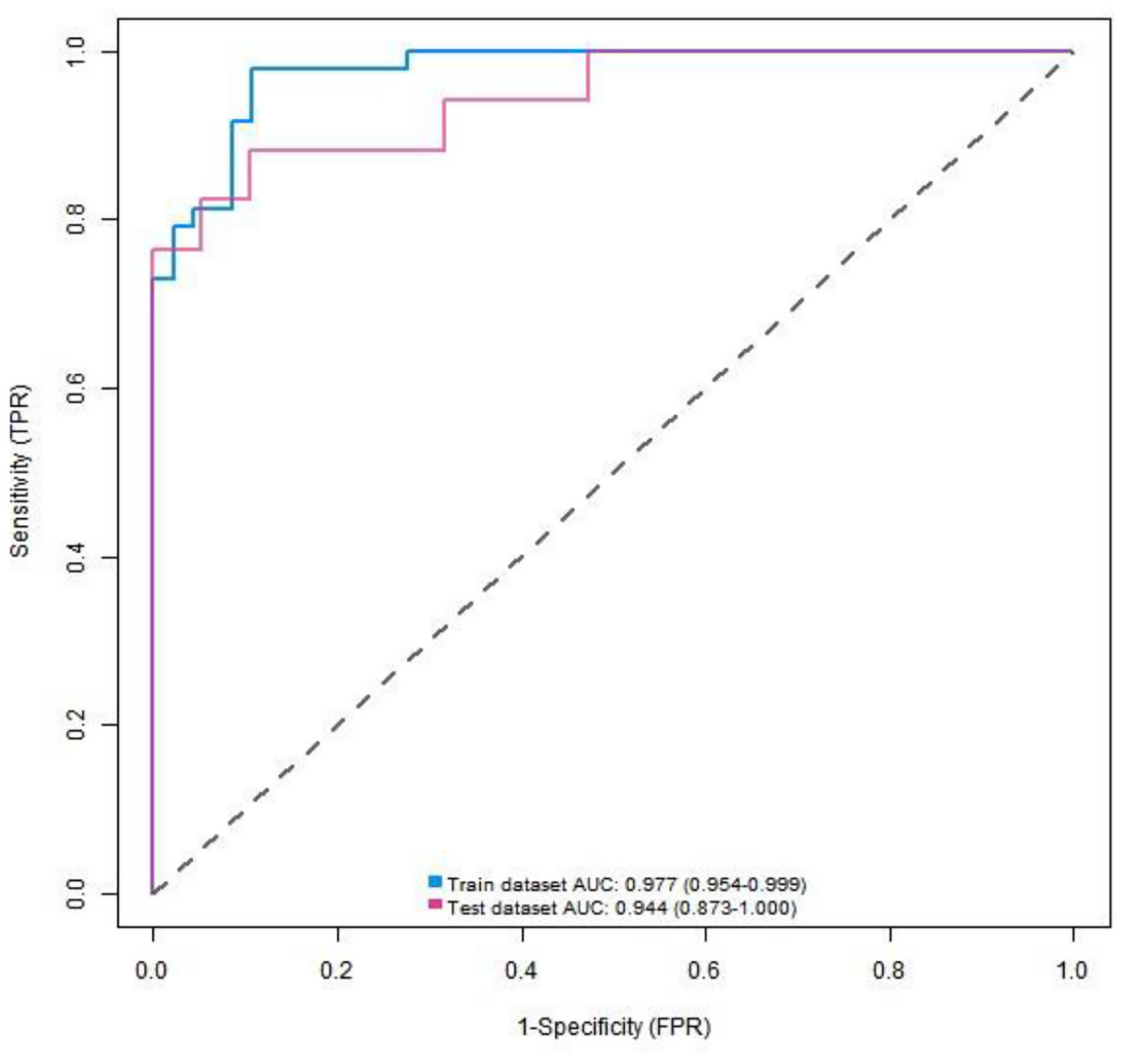
ROC curves of the nomogram model for predicting the risk of hemoptysis in training and validation sets. The area under the curve (AUC) values of 0.977 and 0.944 indicate excellent discriminative ability in both cohorts. FPR = false positive rate, ROC = receiver operating characteristic, TPR = true positive rate.

Discrimination: The nomogram demonstrated strong discriminative ability in both the training and validation sets. In the training set, the AUC was 0.977 (95% CI: 0.954–0.999), while in the validation set, the AUC reached 0.944 (95% CI: 0.873–1.000; Fig. 3). The consistently high AUC values across the 2 independent cohorts strongly verified the outstanding predictive accuracy and robust generalizability of the model.

**Figure 4.**
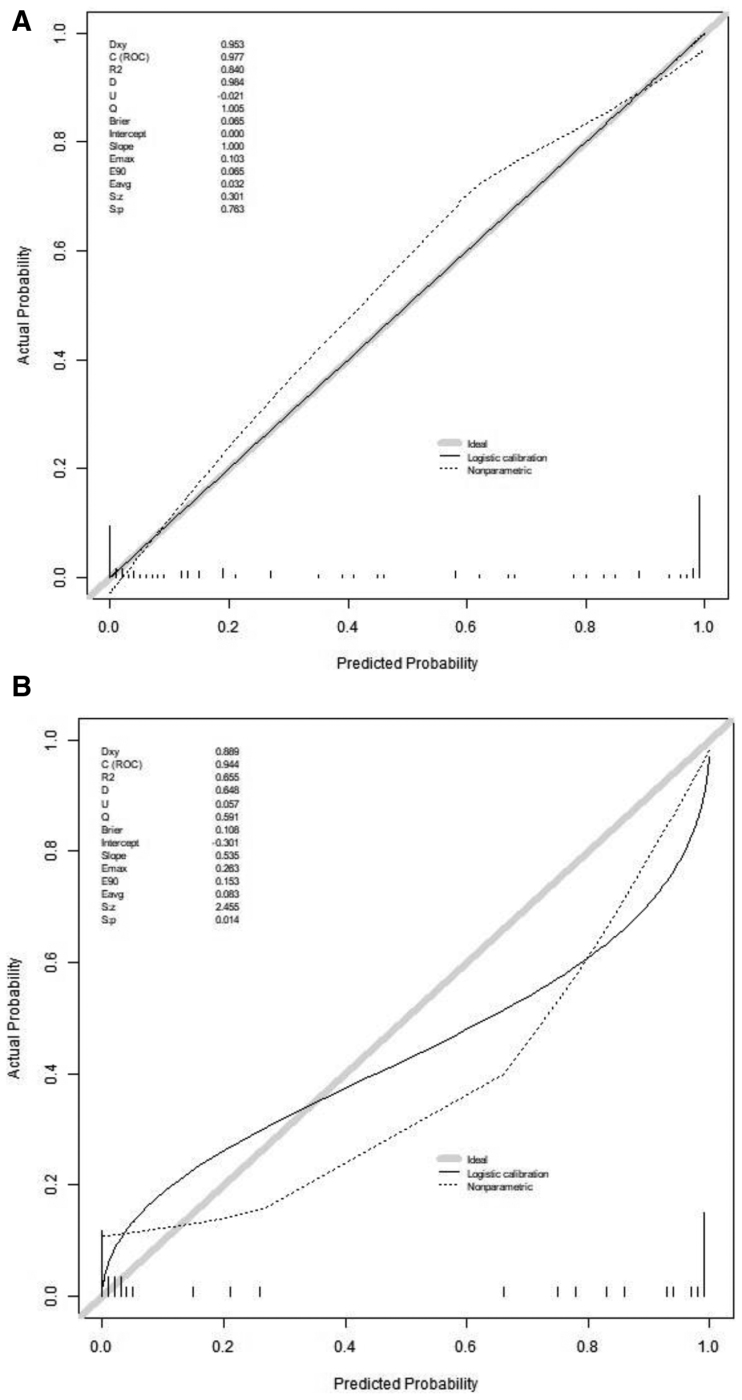
Calibration curves of the nomogram model in the training set (A) and validation set (B). The horizontal axis represents the model-predicted hemoptysis risk, and the vertical axis represents the actual observed hemoptysis rate. The diagonal line indicates perfect prediction. The close fitting of the curve to the diagonal demonstrates excellent calibration consistency. ROC = receiver operating characteristic.

Calibration: The calibration curves for both sets closely approximated the 45-degree ideal reference line (Fig. 4). The Hosmer–Lemeshow test yielded nonsignificant *P*-values (training set: *P* = .740; validation set: *P* = .384), confirming favorable calibration and excellent consistency between model-predicted risk and actual observed hemoptysis events.

**Figure 5.**
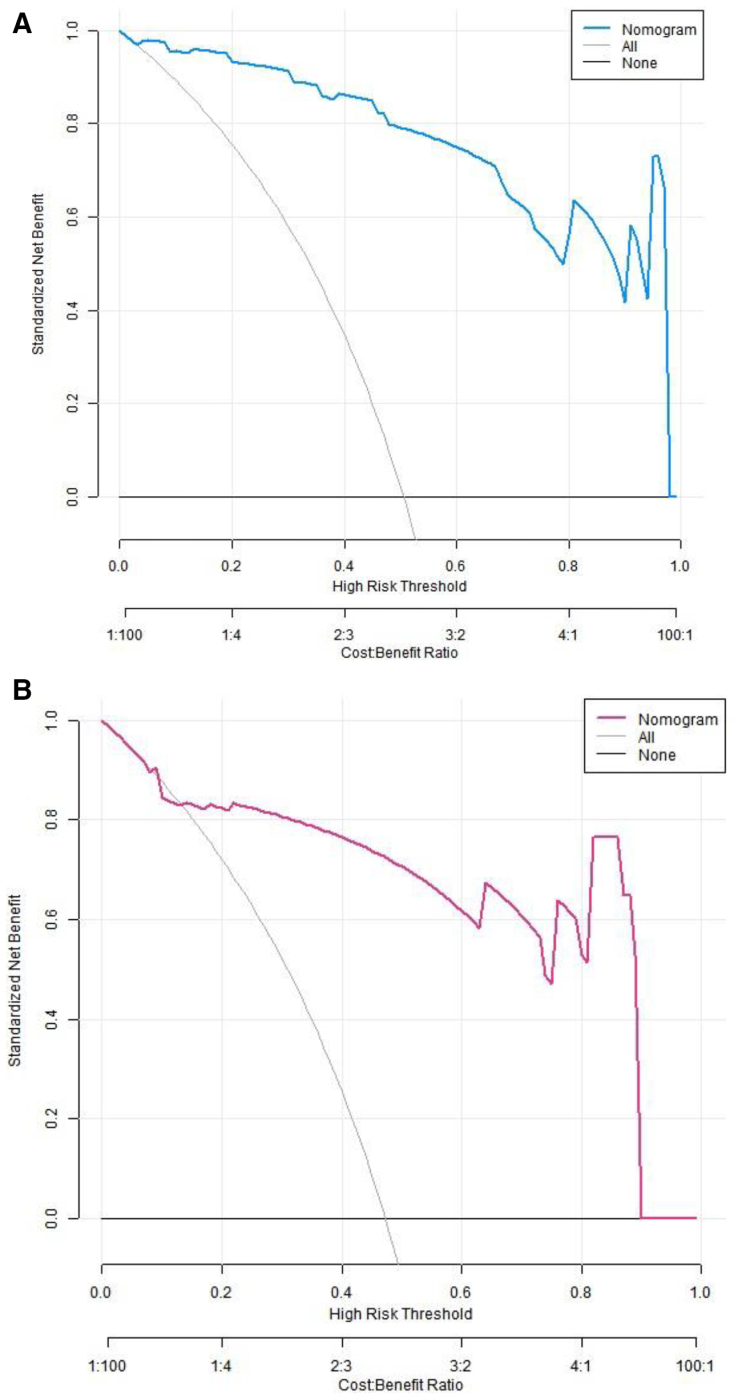
Decision curve analysis (DCA) of the nomogram model in the training set (A) and validation set (B). The red curve represents the net clinical benefit of the nomogram model. The horizontal black line represents the “treat-none” strategy, and the gray line represents the “treat-all” strategy. The model maintained superior net benefit across threshold probability of 15% to 90%.

**Figure 6.**
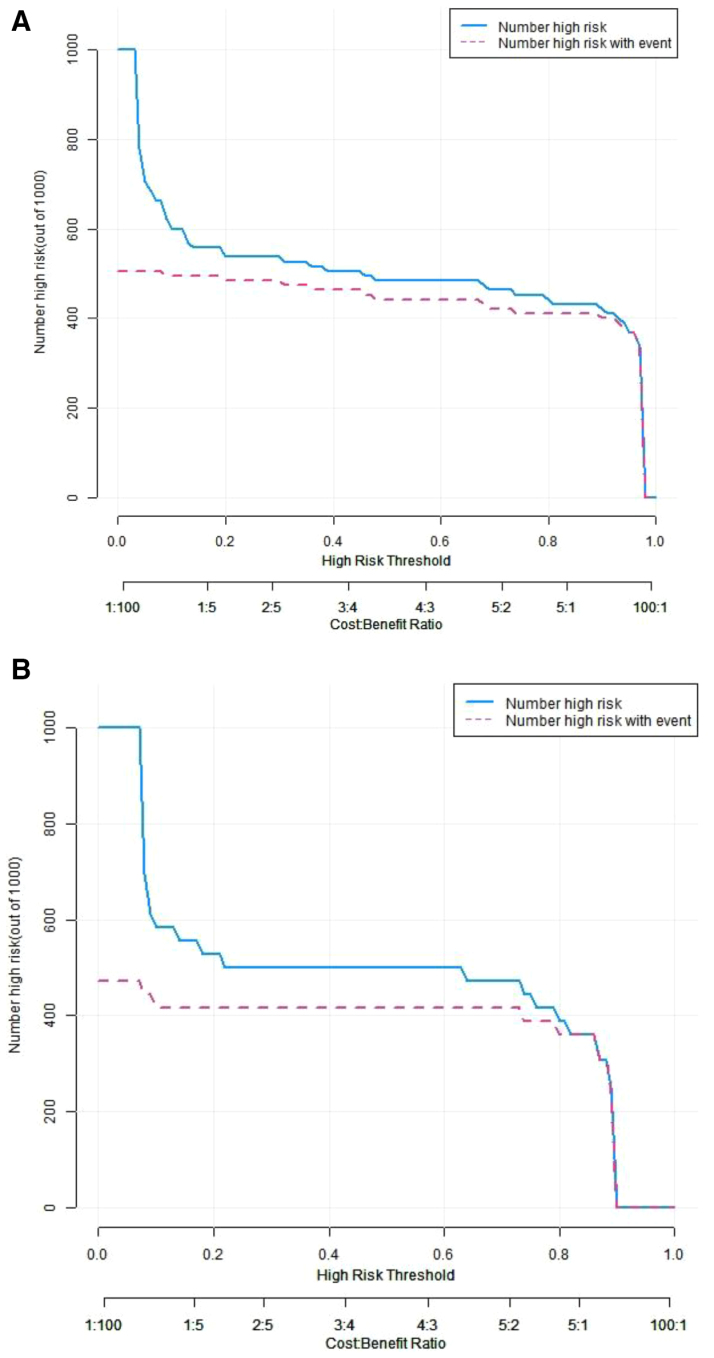
Clinical impact curves (CIC) of the nomogram model in the training set (A) and validation set (B). The curves illustrate the number of high-risk patients screened by the model and the corresponding true-positive cases at different risk thresholds, verifying the reliable clinical risk stratification capability of the nomogram.

Clinical utility: Beyond conventional statistical performance evaluation, clinical utility analysis was performed to verify the translational value of the model in clinical practice. DCA results (Fig. 5) indicated that within a wide clinical threshold probability range (approximately 15%–90%), the nomogram-guided clinical decision-making strategy achieved superior net clinical benefit compared with both the blanket “treat-all” and “treat-none” strategies, demonstrating reliable clinical application value. In addition, the CIC analysis (Fig. 6) further illustrated the model’s excellent risk stratification capability. At a clinically practical risk threshold of 80%, most patients classified as high-risk by the nomogram had true hemoptysis events, with a controllable false-positive rate. This finding indicates that the model can accurately screen high-risk populations while avoiding excessive clinical intervention, further supporting its promising prospect for individualized clinical management and targeted prevention of hemoptysis in bronchiectasis patients.

## 4. Discussion

In this study, we successfully developed and validated a nomogram for predicting hemoptysis risk in patients with bronchiectasis using the Random Forest algorithm. Our data-driven variable screening identified 10 candidate predictive variables, among which 5 variables, including age, BMI, creatinine, CRP, and urea, were confirmed as independent significant predictors of hemoptysis events via multivariate logistic regression analysis after parameter correction (all *P* < .05). The resulting model demonstrated excellent predictive performance in both the training and validation sets, with AUCs of 0.977 and 0.944, respectively. Calibration curves and decision curve analyses further confirmed the model’s high accuracy and clinical utility.

The primary innovation of our study lies in the application of the Random Forest machine learning algorithm for variable selection. Unlike traditional a priori variable selection methods used in logistic regression, the MDA metric of the Random Forest algorithm objectively quantifies each variable’s independent contribution to the model’s predictive accuracy. This approach effectively mitigates investigator selection bias and captures complex nonlinear relationships between variables.^[20]^ Notably, the final set of predictors, particularly BMI, CRP, and creatinine, does not consist entirely of conventional parameters routinely assessed by pulmonologists when evaluating hemoptysis risk. This highlights the powerful capability of machine learning methods to identify potential novel predictors.^[21]^ Our results suggest that the pathophysiological mechanism of hemoptysis in bronchiectasis extends beyond the traditional understanding of local airway anatomy and infection, potentially representing a systemic process closely associated with systemic inflammation, nutritional-metabolic status, and comorbidities.^[22]^

In-depth interpretation of each independent predictor is crucial. First, low BMI emerged as one of the strongest predictors, consistent with previous studies. Malnutrition is a common extrapulmonary manifestation in patients with bronchiectasis; low BMI reflects disease severity and a systemic catabolic state, potentially leading to respiratory muscle weakness, impaired immune function, and reduced tissue repair capacity, thereby indirectly increasing hemoptysis risk.^[23]^ Second, the significance of elevated CRP levels as a marker of systemic inflammation is self-evident. Persistent, high-intensity inflammatory responses erode bronchial vessel walls, leading to neovascularization and the formation of fragile, rupture-prone vascular plexuses, which are the direct pathological basis for hemoptysis.^[24,25]^ Third, creatinine and urea levels collectively suggest that renal function may be a previously overlooked critical factor. Renal insufficiency can cause uremic platelet dysfunction and coagulation pathologies, potentially predisposing patients to coagulation disorders that, once airway bleeding occurs, make spontaneous hemostasis more difficult.^[26,27]^ Additionally, advancing age is a natural risk factor for many chronic diseases and may be associated with increased vascular fragility and multiple comorbidities.^[28]^ Notably, although urea was screened as a candidate variable by machine learning, it failed to maintain independent predictive significance after adjustment for confounding factors, indicating that creatinine is the more stable and reliable renal-related predictive indicator for bronchiectasis-related hemoptysis in this population.

The exceptional discriminative ability (AUC > 0.94) demonstrated by our nomogram model is rarely achieved in clinical prediction models, which likely stems from the advanced algorithm and the highly predictive set of variables used. Such high AUC values indicate that the model can almost perfectly distinguish between patients who will and will not experience hemoptysis. Furthermore, the nonsignificant Hosmer–Lemeshow test result and the calibration curve, closely adhering to the diagonal, confirm that the predicted probabilities are highly accurate and reliable.^[29]^ Finally, DCA validated its clinical value: across a very wide range of threshold probabilities (15%–90%), using this model to guide clinical decisions yielded a positive net benefit. This suggests that regardless of whether clinicians adopt an aggressive or conservative intervention strategy, that is, regardless of the probability threshold they consider worthy of action, the model provides valuable guidance. It thus extends beyond mere statistical performance and holds significant potential to influence clinical practice.^[30]^

However, this study has several limitations. First, as a single-center retrospective investigation, although internal validation was performed, the model still requires further validation in prospective, multicenter external cohorts to establish its generalizability. Second, the sample size was relatively small, particularly in the validation set. Although the results were significant, studies with larger samples would yield more precise estimates. Third, several known or suspected predictors of hemoptysis, such as specific morphological types of bronchiectasis (cylindrical and cystic), detailed distribution of lesions, and comprehensive evaluation of pulmonary hypertension, were not included in this analysis. Future studies should incorporate these variables to explore their contributions. Finally, although we made the model interpretable through a nomogram, machine learning models are often regarded as “black boxes.” The underlying complex interactions captured by the model still require further basic research to be fully elucidated.

In summary, this study employed a Random Forest algorithm to identify the important roles of systemic inflammation, nutritional status, and renal function in hemoptysis among patients with bronchiectasis. Based on these insights, we developed a high-performance, individualized predictive nomogram. After correcting the statistical parameter transcription errors in the logistic regression model, the model not only demonstrated excellent predictive accuracy but also provided a clinical net benefit, providing clinicians with a powerful and user-friendly tool for the early identification of high-risk patients and the implementation of personalized monitoring and preventive strategies. Future research should focus on external validation in prospective multicenter cohorts and explore the feasibility of integrating such models into clinical decision support systems.

## Author contributions

**Conceptualization:** Gengyan Zhang, Lianbo Zhao.

**Formal analysis:** Yan Chen.

**Data curation:** Ming Zhai.

**Writing – original draft:** Gengyan Zhang.

**Writing – review & editing:** Gengyan Zhang, Lianbo Zhao.
